# Pubic Lice in Facial Hair

**DOI:** 10.5826/dpc.1002a42

**Published:** 2020-04-20

**Authors:** Puravoor Jayasree, Feroze Kaliyadan, Karalikkattil T. Ashique

**Affiliations:** 1 Dermatology, Medical Trust Hospital, Cochin, Kerala, India; 2 Dermatology, College of Medicine, King Faisal University, Saudi Arabia; 3 Amanza Health Care, Nahas Skin Clinic, Perinthalmanna, Kerala, India

**Keywords:** lice infestations, pruritus, hair, *Phthirus*, dermoscopy

## Case Presentation

**Figure 1:**
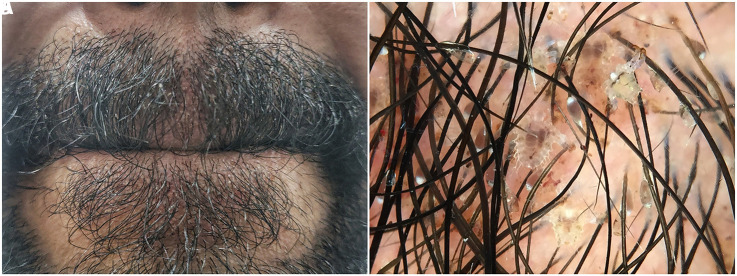
(A) Facial hair showing yellowish deposits and erythema of underlying skin. (B) Crab- shaped pubic lice with thick claws grasping hair shafts along with brown (viable) and translucent (empty) nits seen on polarized dermoscopy.

A single male in his twenties presented with itching and a foreign body sensation in his moustache. He reported active, oral sexual contact with a female acquaintance 3 days prior to the onset of symptoms. Examination revealed yellowish deposits on the moustache and facial hair below the lower lip with erythema of the underlying skin (Figure 1A). On dermoscopy, crab-shaped parasites with broad bodies and thick claws were spotted clutching the hair shafts. Viable nits containing unhatched nymphs and empty translucent nit casings were also seen attached to proximal hair shafts (Figure 1B). Examination of the remaining hair-bearing skin including pubic area and eyelashes did not yield any findings. Thus a diagnosis of phthiriasis of the facial hair was made.

## Teaching Point

*Phthirus pubis* or pubic louse can infest and survive in any hair-bearing skin [1]. Entomodermoscopy serves as a rapid diagnostic tool [2].
